# Adaptive Response of *Listeria monocytogenes* to Heat, Salinity and Low pH, after Habituation on Cherry Tomatoes and Lettuce Leaves

**DOI:** 10.1371/journal.pone.0165746

**Published:** 2016-10-31

**Authors:** Sofia V. Poimenidou, Danai-Natalia Chatzithoma, George-John Nychas, Panagiotis N. Skandamis

**Affiliations:** 1 Laboratory of Food Quality Control and Hygiene, Department of Food Science and Human Nutrition, Agricultural University of Athens, Iera Odos 75, 118 55, Athens, Greece; 2 Laboratory of Food Microbiology and Biotechnology, Department of Food Science and Human Nutrition, Agricultural University of Athens, Iera Odos 75, 118 55, Athens, Greece; University of Copenhagen, DENMARK

## Abstract

Pathogens found on fresh produce may encounter low temperatures, high acidity and limited nutrient availability. The aim of this study was to evaluate the effect of habituation of *Listeria monocytogenes* on cherry tomatoes or lettuce leaves on its subsequent response to inhibitory levels of acid, osmotic and heat stress. Habituation was performed by inoculating lettuce coupons, whole cherry tomatoes or tryptic soy broth (TSB) with a three-strains composite of *L*. *monocytogenes*, which were further incubated at 5°C for 24 hours or 5 days. Additionally, cells grown overnight in TSB supplemented with 0.6% yeast extract (TSBYE) at 30°C were used as control cells. Following habituation, *L*. *monocytogenes* cells were harvested and exposed to: (i) pH 3.5 adjusted with lactic acid, acetic acid or hydrochloric acid (HCl), and pH 1.5 (HCl) for 6 h; (ii) 20% NaCl and (iii) 60°C for 150 s. Results showed that tomato-habituated *L*. *monocytogenes* cells were more tolerant (*P* < 0.05) to acid or osmotic stress than those habituated on lettuce, and habituation on both foods resulted in more stress resistant cells than prior growth in TSB. On the contrary, the highest resistance to heat stress (*P* < 0.05) was exhibited by the lettuce-habituated *L*. *monocytogenes* cells followed by TSB-grown cells at 5°C for 24 h, whereas tomato-habituated cells were highly sensitized. Prolonged starvation on fresh produce (5 days vs. 24 h) increased resistance to osmotic and acid stress, but reduced thermotolerance, regardless of the pre-exposure environment (i.e., tomatoes, lettuce or TSB). These results indicate that *L*. *monocytogenes* cells habituated on fresh produce at low temperatures might acquire resistance to subsequent antimicrobial treatments raising important food safety implications.

## Introduction

Fresh fruits and vegetables are an integral part of a healthy diet, rich in nutrients, fibers and vitamins. Nevertheless, during the last decades they have also been recognized as a potential vehicle of foodborne pathogens. Numerous studies have reported that fresh produce might be implicated in foodborne outbreaks, as contamination by pathogenic microorganisms may occur in the field, during post-harvest processing or the handling in domestic environments [1–3]. *Listeria monocytogenes* is a foodborne pathogen, able to survive under a wide range of environmental conditions (pH, temperature, and a_w_) [4]. It is the causative agent of listeriosis, a severe foodborne disease with high mortality rates among the immune-compromised individuals, pregnant women, neonates, and the elderly. Due to its widespread occurrence in the environment, ability to attach to surfaces and tolerance to stress factors, the incidence rate of *L*. *monocytogenes* in fresh produce and the food-processing environment is remarkably high [5–8]. In 2014, data provided by 15 EU member states for 3.272 units of ready-to-eat (RTE) fruit and vegetables showed that 2.8% were positive for *L*. *monocytogenes* detection [9].

Studies evaluating stress responses have shown that exposure of *L*. *monocytogenes* to a sublethal stress may induce adaptive responses to subsequent lethal stress [10–13]. In addition, the ecological background of *L*. *monocytogenes* cells could potentially affect their subsequent physiological behavior [14–17]. The fresh produce matrix is a complex ecological niche, generally considered as a hostile environment, where epiphytic fitness of pathogenic bacteria is dependent on their interactions with resident microbiota, utilization of available nutrients and spatial heterogeneity in physicochemical conditions (e.g. pH, water availability, osmotic stress, etc.) [18,19]. Despite the efforts made towards the elimination of pathogens from minimally processed vegetables [20], they manage to survive, which could be partly attributed to acquired stress tolerance via adaptation mechanisms. Various types of vegetables support growth or the survival of *L*. *monocytogenes* that may originate from the raw materials or the processing environment, e.g., attached on shredders, cutting boards or blades used for preparation of fresh cut salads [21–24]. These in combination with the ability of *L*. *monocytogenes* to grow during refrigerated storage are likely contributing factors to the increasing trend of listeriosis associated with consumption of RTE foods [25]. Consequently, whether and how habituation or growth of *L*. *monocytogenes* on fresh cut salads impacts the stress tolerance phenotype of the organism to food processing- or host-related stresses (e.g., gastric acidity) is worth investigating for assessing the potential food safety threat along the supply chain of fresh produce.

Considering the above, the objective of the present study was to evaluate the tolerance of *L*. *monocytogenes* cells against heat, osmotic or acid conditions, upon their habituation on lettuce or cherry tomatoes, at 5°C for 24 h or 5 days. The food models were selected to represent different pH, nutritional and surface characteristics, and from an exposure assessment perspective to represent products of high consumption frequency, also implicated in recent foodborne disease outbreaks [26].

## Materials and Methods

### *Listeria monocytogenes* strains and inoculum preparation

Three strains of *Listeria monocytogenes* (strain C5 serotype 4b, strain 6179 serotype 1/2a and strain Scott A serotype 4b) were used in this study. Strains C5 and 6179 were kindly provided by Dr. K. Jordan (Teagasc, Fermoy, Ireland). Stock cultures were stored at -20°C in tryptic soy broth (TSB; Lab M Limited, United Kingdom) supplemented with 20% (v/v) glycerol. Culture slants were prepared for each *L*. *monocytogenes* strain individually, cultivated on tryptic soy agar (TSA; Lab M Limited, United Kingdom) supplemented with 0.6% (w/v) yeast extract (YE; Lab M Limited, United Kingdom), and stored at 4°C for up to thirty days. Prior to experimentation, each strain was subcultured twice in TSB-YE at 30°C, for 24 h and 18 h, respectively. The 18-h activated strain cultures were harvested individually by centrifugation at 3600 rpm, 15 min, 4°C (Megafuge1.0 R, Heraeus Instruments), resuspended in 10 mL of ¼ Ringer solution (Ringer’s solution tablet; Lab M Limited, United Kingdom) and mixed at equal volumes of 10 mL. The mixed inoculum was decimally diluted and used for the inoculation of fresh produce.

### Habituation on fresh produce and *in vitro* controls

*L*. *monocytogenes* cells were subjected to the following culture preparation scenarios prior to exposure to inhibitory stresses: (i) habituation on tomatoes or lettuce for 24 h or 5 days, at 5°C and (ii) growth in TSB cells for 24 h or 5 days, at 5°C. Cells grown in TSB overnight, at 30°C, were considered as *in vitro* control. Incubation time of 24 h was based on studies that evaluated the antimicrobial efficacy of decontamination washings on contaminated by pathogens fresh produce [27–30].

Romaine lettuce (*Lactuca sativa*) and cherry tomatoes (*Solanum lycopersicum* var. *cerasiforme;* hereafter referred to as tomatoes) were purchased from a local market (Athens, Greece) on the experimentation day, washed thoroughly with tap water and placed on a sterile tray for 15 min for drying. Lettuce leaves were cut in rectangular pieces of 4 × 2 cm, and portions of 10 g were placed into sterile stomacher bags. An aliquot (1 mL) of the inoculum (~10^7^ CFU/mL) was added per bag and dispersed carefully by gentle massaging to achieve homogeneous distribution. Tomatoes were immersed into a glass beaker containing 200 mL of inoculum (~10^7^ CFU/mL) manually agitated for 2 min and transferred into a stomacher bag. Bags containing the inoculated lettuce or tomatoes samples were incubated for 24 h or 5 days at 5°C. Additionally, sterile plastic tubes containing 10 mL TSB were inoculated with 0.1 mL of *L*. *monocytogenes* inoculum, to a final level of ~10^5^ CFU/mL, and were incubated for 24 h or 5 days, at 5°C. Experiments were carried out in duplicate with three technical replicates each.

### Harvest of habituated *L*. *monocytogenes*

Cells habituated on lettuce or tomatoes at 5°C for 24 h or 5 days were harvested as follows: produce samples were removed from the bags and washed under mild agitation in 300 mL of Ringer solution for 20 s, in order to remove loosely attached cells (approximately 0.7 log CFU/g). Inoculated samples were then transferred to filter stomacher bags, mixed with new Ringer solution at 1:5 (w/v) ratio and homogenized in stomacher for 30 s. An aliquot of 100 μL from the bag or from inoculated TSB tubes was decimally diluted and enumerated. Following the aforementioned habituation or growth scenarios, in order to carry out the challenge tests against inhibitory stresses, bacterial populations from food samples or TSB were harvested by centrifugation (3600 rpm, 5 min, 5°C), washed and resuspended in 3 mL Ringer to be further challenged.

### Challenge against inhibitory stresses

Adaptive response of cell cultures was evaluated against inhibitory acid, heat and osmotic conditions. Sterile TSB was adjusted to pH 3.5 with lactic acid (Fluka, St. Louis, USA; 0.12 M), acetic acid (Panreac, Barcelona, Spain; 0.62 M) or hydrochloric acid (HCl; Panreac), or to pH 1.5 with HCl. The undissociated acid concentration (UAC) of the organic acids was calculated according to the Henderson–Hasselbalch equation: UAC = TAC / (1+10^pH-pKa^), where pH is the pH value of the solution and TAC is the total acid concentration. In each case, acidified TSB was distributed in aliquots of 27 mL into 50-mL sterile plastic tubes. Aliquots (3 mL) of cell suspensions, prepared as described above, were added to each plastic tube in triplicate, so as the initial pathogen population exposed to acid was approximately 10^5^ cells/mL. The tubes were incubated at 25°C (ambient temperature) for 6 hours. Every hour, 1 mL was removed for enumeration of surviving population after proper serial decimal dilutions and plating, as described below.

In order to assess the osmotolerance of the habituated cells, 27 mL volumes of TSB containing 20% NaCl (w/v) were distributed to sterile 50-mL plastic tubes and inoculated with 3 mL of cell suspension, targeting a final concentration of approximately 10^5^ CFU/mL. Plastic tubes were stored at 25°C and every day samplings took place for as long as surviving populations were quantifiable (detection limit: 1 CFU/mL).

Thermal inactivation of habituated cell cultures was estimated according to the method described by Bacon et al. [31]. Sterile capillary tubes (Vitrex® Micro haematocrit tubes, Denmark; 1.15 to 1.55 by 75mm) were filled with 0.05 mL of cell suspension, and were manually heat sealed with a propane torch carefully, to avoid heating of the cells. Capillary tubes were then heated in a thermostatically controlled circulating water bath for 150 s. Every 30 s, tubes were removed from the water bath, immediately cooled in ice-water and sanitized in sodium hypochlorite solution (NaOCl Merck, Darmstadt, Germany, active chlorine 6–14%; 200 ppm, pH 6.5). The tubes were then rinsed with sterile deionized H_2_O, cracked, and the content was dispensed in eppendorff tubes containing Ringer solution. Aliquots (100 μL) of proper decimal dilutions were plated for cell enumeration. All assays were performed in duplicate and three independent samples were examined during each challenge experiment.

Enumeration of *L*. *monocytogenes* population was carried out on PALCAM (Lab M Limited, United Kingdom) and TSAYE media, following incubation at 30°C for 48 h. No significant differences (*P* > 0.05) were observed among stressed populations enumerated on selective and non-selective media.

### Data fitting

Inactivation kinetic parameters for acid, osmotic and heat challenge were determined by fitting the log transformed levels of surviving populations to Weibull model [32], of the following form: log *N/N*_*0*_ = -(*t/δ*)^*p*^, where *N* is the population (CFU/mL) of *L*. *monocytogenes* at time *t*, and *N*_*0*_ is the initial population at *t*_*0*_; *δ* is the time (min, days or s, respectively) till the first decimal reduction; and *p* is a shape parameter, corresponding to upward concavity of the survival curve (*p* < 1), linear survival curve (*p* = 1) or downward concavity (*p* > 1). For the heat challenge inactivation curves, the modified Weibull model of double sigmoidal inactivation [33] for cells previously habituated on lettuce (for 24 h and 5 days), and the modified Weibull model which includes the curve tailing [34], for cells previously habituated on tomatoes for 24 h, were used. The goodness-of-fit of the models was evaluated using coefficient correlation (*R*^*2*^) and root-mean square error (RMSE). Data fitting was performed using the software GInaFiT, a freeware Add-in for Microsoft® Excel (available at http://cit.kuleuven.be/biotec/ginafit.php).

### Statistical analysis

Cell populations were transformed to log_10_ CFU/mL and expressed as % ratio ([(final CFU/mL) / (initial CFU/mL)]×100) in 30 mL TSB that contained the stress factor. Detection limit was 1 CFU/mL for cultures subjected to acid or osmotic stress, and 20 CFU/mL for those subjected to heat stress. Analysis of variance (ANOVA) and Tukey ‘s honestly significant differences (HSD) test (*P* < 0.05) were performed to estimate significant differences within each fitting parameter or among cell populations for each time point during stress tests, and *t* test for differences between 24-h and 5-day habituation, by using JMP 9 Statistical Software (SAS Institute, Cary, NC).

## Results

### Microbial populations before stress challenge

**Fig 1:**
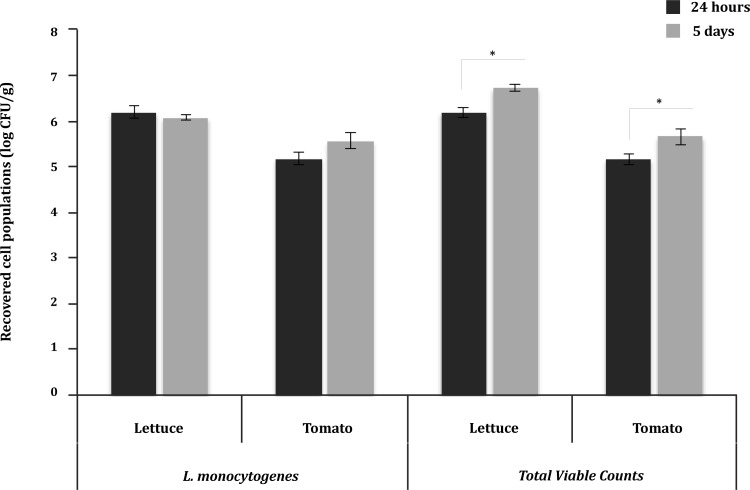
Growth of *L*. *monocytogenes* and total microbiota on fresh produce. Size of populations (log CFU/g) of *L*. *monocytogenes* and total microbiota on inoculated lettuce and tomatoes were determined following 24-h and 5-day storage at 5°C. Bars represent mean populations ± standard error mean of eight independent experiments with three technical replicates each (n = 24). Significant differences (*P* < 0.05) between 24-h and 5-day stored samples are shown as (*).

Populations of *L*. *monocytogenes* and total viable counts recovered from inoculated lettuce and tomatoes, after 24 h or 5 days of incubation at 5°C, are illustrated on Fig 1. No significant changes in pathogen populations were observed, while total viable counts increased from 6.2 to 6.7 log CFU/g on lettuce and from 5.2 to 5.7 log CFU/g on tomatoes (*P* < 0.05). In TSB stored at 5°C, *L*. *monocytogenes* population increased between 24 h and 5 days of incubation by 1.3 log CFU/mL; the pH values were 7.4 and 7.3, respectively.

### Acid response of *L*. *monocytogenes*

**Fig 2:**
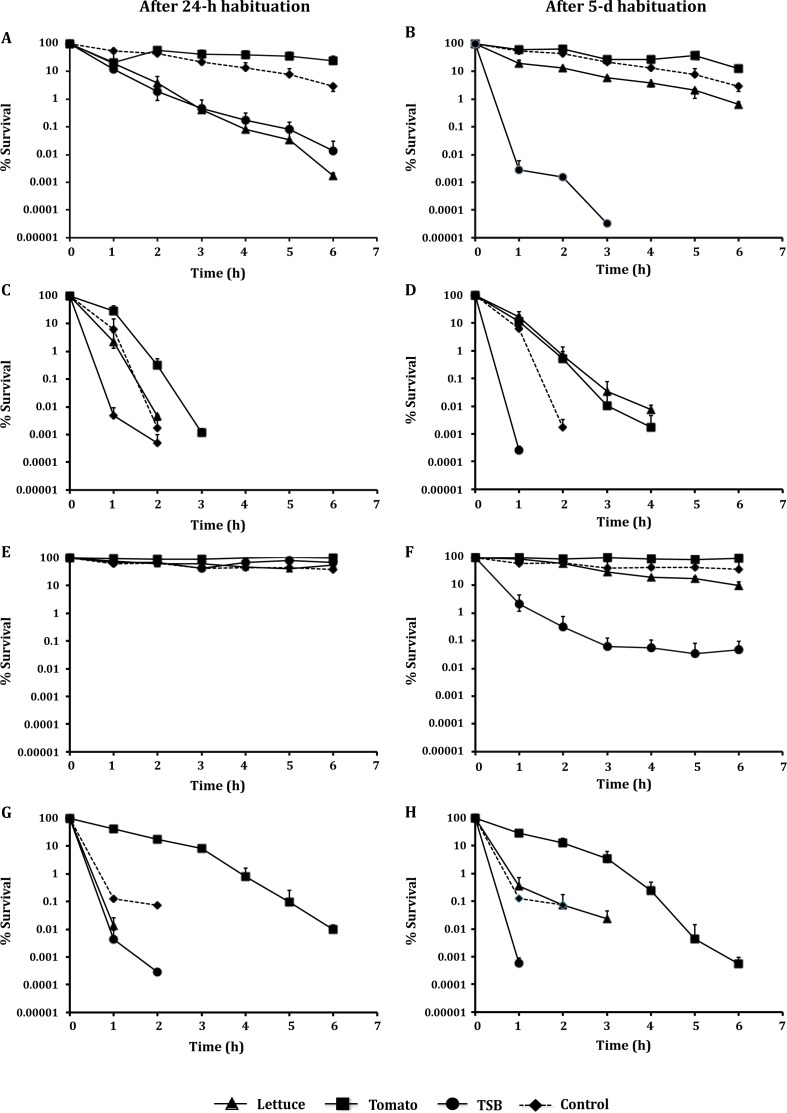
*L*. *monocytogenes* acid stress survival after habituation on fresh produce. Survival (%) of *L*. *monocytogenes* population was determined after exposure to lactic acid (A-B); acetic acid (C-D); HCl at pH 3.5 (E-F); and HCl at pH 1.5 (G-H), for 6 h. Cell cultures were previously habituated on lettuce, tomatoes, or in TSB, at 5°C for 24 h and 5 days, and in TSB for 18 h at 30°C (control). Values represent the mean of two independent experiments, with 3 technical replicates each (n = 6).

During the 6-h exposure to lactic acid (pH 3.5), significant variability in acid resistance was observed for cell cultures previously habituated on different environments (Fig 2A and 2B). The most tolerant cells (*P* < 0.05) were those habituated on tomatoes (higher *δ* values; i.e., time of the first decimal reduction), and specifically, cells following the 5-day habituation, which exhibited the highest *δ*-values (Table 1). After 6 hours of acid challenge, higher surviving populations (N_final_) were observed for tomato-habituated cells (24.8% of the initial population of 24-h cells and 12.6% of 5-day cells) and control cells (TSB 18 h, 30°C; 2.9%). Regarding lettuce, 5-day habituation resulted in higher tolerance than the 24-h habituation, as expressed by *t*_4D_ (i.e., time to 4 log reductions of the initial microbial population), with 5-day habituated pathogen surviving for longer than 360 min compared to 24-h cells, which were inactivated after 265 min_._ Among all cell cultures, the least tolerance was exhibited by cells habituated in TSB at 5°C, where 5-day habituation resulted in the shortest *t*_4D_ value (Table 1).

**Table 1:** Estimates of Weibull model for inactivation curves during acid challenge, using lactic acid, for 6 h at 25°C.

*Habituation*		Lactic acid pH 3.5				
Environment	Conditions	*δ (min to first decimal reduction)*	*p (shape parameter)*	*t*_*4D*_ *(min to 4-log reduction)*	*R*^*2*^	*RMSE*
Lettuce	24 h, 5°C	92.8 ± 18.6^D^	1.4 ± 0.3^A^	265.6 ± 69.5^A^	0.99 ± 0.00	0.252 ± 0.024
	5 days, 5°C	129.5 ± 12.2^CD^	0.6 ± 0.1^A^	> 360	0.99 ± 0.01	0.077 ± 0.197
Tomatoes	24 h, 5°C	408.6 ± 3.3^ΑΒ^	5.2 ± 8.6^A^	> 360	0.43 ± 0.41	0.309 ± 0.080
	5 days, 5°C	523.7 ± 139.8^Α^	0.8 ± 0.2^A^	> 360	0.77 ± 0.19	0.166 ± 0.098
TSB	24 h, 5°C	49.2 ± 14.9^D^	0.7 ± 0.1^A^	319.2 ± 22.0^A^	0.99 ± 0.01	0.214 ± 0.132
	5 days, 5°C	27.9 ± 31.2^D^	1.4 ± 1.2^A^	77.4 ± 25.2^B^	0.99 ± 0.01	0.491 ± 0.017
	18 h, 30°C	261.9 ± 49.5^BC^	1.5 ± 0.7^A^	> 360	0.97 ± 0.01	0.106 ± 0.000

Values represent mean ± stdev of six replicates. Different letters within the same column indicate significantly different (*P* < 0.05) values of each parameter.

*R*^*2*^: regression coefficient

RMSE: Root-mean square error

Regarding the acetic acid (pH 3.5), all *L*. *monocytogenes* cultures survived no longer than 4 h (Fig 2C and 2D). *p* values (i.e., shape of inactivation curve) were close to 1 and inactivation curves had a linear behavior. Cells previously habituated on tomatoes for 24 h exhibited significantly higher tolerance, as expressed by *δ* values (min), compared to the cell cultures habituated in TSB at 5°C (Table 2). Habituation on lettuce for 5 days strengthened the cells compared to 24-h lettuce cells, resulting in higher *t*_4D_ (*P* < 0.05); these cells were also more resistant (higher *t*_4D_) compared to TSB-habituated cells.

**Table 2:** Estimates of Weibull model for inactivation curves during acid challenge, using acetic acid, for 6 h at 25°C.

*Habituation*		Acetic acid pH 3.5				
Environment	Conditions	*δ (min to first decimal reduction)*	*p (shape parameter)*	*t*_*4D*_ *(min for 4-log reduction)*	*R*^*2*^	*RMSE*
Lettuce	24 h, 5°C	34.6 ± 18.3^AB^	1.3 ± 0.3^A^	97.5 ± 36.8^CD^	1.00 ± 0.00	0.000 ± 0.174
	5 days, 5°C	61.2 ± 20.2^AB^	1.2 ± 0.4^A^	203.7 ± 41.2^A^	0.99 ± 0.01	0.181 ± 0.250
Tomato	24 h, 5°C	76.2 ± 8.3^A^	1.6 ± 0.3^A^	184.0 ± 38.1^AB^	0.99 ± 0.01	0.270 ± 0.126
	5 days, 5°C	66.9 ± 10.2^AB^	1.5 ± 0.1^A^	166.5 ± 16.6^ABC^	0.99 ± 0.00	0.167 ± 0.558
TSB	24 h, 5°C	28.6 ± 21.7^B^	1.4 ± 0.7^A^	82.0 ± 31.8^CD^	0.99 ± 0.02	0.279 ± 0.000
	5 days, 5°C	11.3 ± 0.3^B^	1.0 ± 0.0^A^	55.0 ± 0.0^D^	0.99 ± 0.00	0.000 ± 0.576
	18 h, 30°C	48.6 ± 15.5^AB^	1.7 ± 0.6^A^	117.8 ± 2.4^BCD^	0.98 ± 0.02	0.479 ± 0.576

Values represent mean ± stdev of six replicates. Different letters within the same column indicate significantly different (*P* < 0.05) values of each parameter.

*R*^*2*^: regression coefficient

RMSE: Root-mean square error

Hydrochloric acid at pH 3.5 was not lethal for any of the cell cultures during the 6-h exposure, as none of them were reduced to below the detection limit (1 CFU/mL) (Fig 2). Parameter estimates could be determined only for the inactivation curves of 5-day lettuce-habituated and TSB-habituated cells (Table 3). The most tolerant cultures (*P* < 0.05) were those habituated on tomatoes for 24 h or 5 days, which at the end of the challenge, were reduced to 96.8% and 94.5% of the initial population, respectively (Fig 2E and 2F). The lowest survival (*P* < 0.05) was observed for cells habituated in TSB for 5 days at 5°C, with *δ* = 13.8 min. Habituation on lettuce for 5 days resulted in weakening of *L*. *monocytogenes*, manifested as a marked decrease in cell population during acid challenge (Fig 2F), contrary to 24-hour cells with no considerable decrease in cell concentration; populations of 24-h and 5-day lettuce-habituated cells were reduced to 53.5% and 9.8%, respectively.

**Table 3:** Estimates of Weibull model for inactivation curves during acid challenge, using HCl, for 6 h at 25°C.

*Habituation*		HCl pH 3.5				
Environment	Conditions	*δ (min to first decimal reduction)*	*p (shape parameter)*	*t*_*4D*_ *(min to 4-log reduction)*	*R*^*2*^	*RMSE*
Lettuce	24 h, 5°C	-	-	-	-	-
	5 days, 5°C	348.7 ± 35.5^Α^	1.1 ± 0.2	> 360	0.99 ± 0.01	0.080 ± 0.021
Tomato	24 h, 5°C	-	-	-	-	-
	5 days, 5°C	-	-	-	-	-
TSB	24 h, 5°C	-	-	-	-	-
	5 days, 5°C	13.8 ± 12.1^Β^	0.5 ± 0.4	> 360	0.99 ± 0.01	0.323 ± 0.050
	18 h, 30°C	-	-	-	-	-

Values represent mean ± stdev of six replicates. Different letters within the same column indicate significantly different (*P* < 0.05) values of each parameter.

*R*^*2*^: regression coefficient

RMSE: Root-mean square error

“-“: Parameters were not determined due to low inactivation rate of the pathogen cells.

Among *L*. *monocytogenes* cultures exposed to HCl pH 1.5 (Fig 2G and 2H), the most tolerant cells were those previously habituated on tomatoes, with higher *δ* and *t*_4D_ among all cell cultures (*P* < 0.05) (Table 4), and higher *p* values (*P* < 0.05), indicating a significantly prolonged resistance to stress. No significant differences were observed for the cells previously habituated in TSB or on lettuce.

**Table 4:** Estimates of Weibull model for inactivation curves during acid challenge, using HCl, for 6 h at 25°C.

*Habituation*		HCl pH 1.5				
Environment	Conditions	*δ (min to first decimal reduction)*	*p (shape parameter)*	*t*_*4D*_ *(min to 4-log reduction)*	*R*^*2*^	*RMSE*
Lettuce	24 h, 5°C	16.0 ± 2.3^Β^	1.0 ± 0.0^B^	55.0 ± 0.0^B^	1.00 ± 0.00	-
	5 days, 5°C	18.9 ± 3.0^Β^	0.8 ± 0.2^B^	79.5 ± 49.0^B^	0.99 ± 0.01	0.118 ± 0.200
Tomato	24 h, 5°C	121.1 ± 100.8^A^	3.0 ± 1.5^A^	295.0 ± 3.5^Α^	0.98 ± 0.01	0.223 ± 0.115
	5 days, 5°C	147.7 ± 32.9^Α^	2.1 ± 0.6^A^	302.0 ± 19.1^A^	0.99 ± 0.01	0.230 ± 0.104
TSB	24 h, 5°C	6.2 ± 5.9^Β^	0.7 ± 0.4^B^	56.3 ± 3.3^B^	1.00 ± 0.00	-
	5 days, 5°C	12.0 ± 0.8^Β^	1.0 ± 0.0^B^	55.0 ± 0.0^B^	1.00 ± 0.00	-
	18 h, 30°C	7.6 ± 6.4^Β^	0.5 ± 0.1^B^	84.8 ± 37.4^B^	0.99 ± 0.02	0.178 ± 0.299

Values represent mean ± stdev of six replicates. Different letters within the same column indicate significantly different (*P* < 0.05) values of each parameter.

*R*^*2*^: regression coefficient

RMSE: Root-mean square error

“-“: Not determined

### Osmotolerance of *L*. *monocytogenes* cells

**Fig 3:**
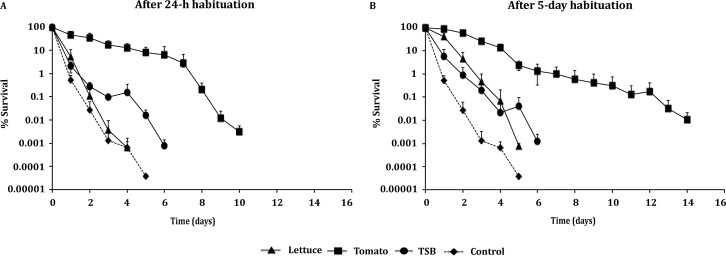
*L*. *monocytogenes* osmotolerance after habituation on fresh produce. Survival (%) of *L*. *monocytogenes* population was determined after exposure to 20% NaCl osmotic stress. Cell cultures were previously habituated on lettuce, tomatoes, or in TSB, at 5°C for 24 h and 5 days, and in TSB for 18 h at 30°C (control). Values represent the mean of two independent experiments, with 3 technical replicates each (n = 6).

The highest osmotolerance was exhibited by cells habituated on tomatoes, with higher *δ* and *t*_4D_ values (*P* < 0.05) than those corresponding to cells from other habituation matrices. Additionally, the higher *p* value (*P* < 0.05) indicated a more prolonged tolerance to the stress for the 24-h tomato habituation (Table 5). Residence in the tomato environment resulted in a longer survival (4-log reduction over 14 days) following 5-day habituation than 24-h habituation (4.5-log reduction over 10 days) (Fig 3). Similar positive effect of storage period occurred for cells habituated on lettuce, where 24-h cells and 5-day cells were inactivated after 4 and 5 days, respectively. No significant differences in *δ* and *t*_4D_ values were observed between the cell cultures habituated on lettuce and in TSB at 5°C.

**Table 5:** Parameter estimates of Weibull model for inactivation curves during osmotic stress, in TSB containing 20% (w/v) NaCl, at 25°C.

*Habituation*		Osmotic stress				
Environment	Conditions	*δ (days to first decimal reduction)*	*p (shape parameter)*	*t*_*4D*_ *(days to 4-log reduction)*	*R*^*2*^	*RMSE*
Lettuce	24 h, 5°C	0.7 ± 0.4^C^	1.0 ± 0.4^B^	2.9 ± 0.4^BC^	0.99 ± 0.01	0.143 ± 0.085
	5 days, 5°C	1.3 ± 0.5^C^	1.1 ± 0.3^B^	4.6 ± 1.0^B^	0.99 ± 0.01	0.313 ± 0.113
Tomato	24 h, 5°C	5.8 ± 1.1^Α^	2.7 ± 0.9^A^	9.9 ± 0.0^A^	0.98 ± 0.01	0.187 ± 0.062
	5 days, 5°C	3.6 ± 0.7^Β^	1.2 ± 0.2^B^	9.0 ± 0.0^A^	0.98 ± 0.01	0.209 ± 0.020
TSB	24 h, 5°C	0.4 ± 0.2^C^	0.6 ± 0.2^B^	5.2 ± 1.0^B^	0.95 ± 0.06	0.350 ± 0.256
	5 days, 5°C	0.8 ± 0.3^C^	0.8 ± 0.0^B^	4.4 ± 1.9^BC^	0.99 ± 0.01	0.132 ± 0.036
	18 h, 30°C	0.3 ± 0.1^C^	0.7 ± 0.2^B^	2.3 ± 0.5^C^	0.98 ± 0.02	0.424 ± 0.253

Values represent mean ± stdev of six replicates. Different letters within the same column indicate significantly different (*P* < 0.05) values of each parameter.

*R*^*2*^: regression coefficient

RMSE: Root-mean square error

### Thermal inactivation of *L*. *monocytogenes* cells

**Fig 4:**
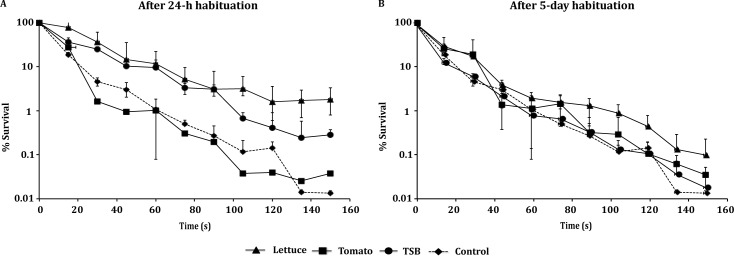
*L*. *monocytogenes* heat response after habituation on fresh produce. Survival (%) of *L*. *monocytogenes* population was determined after exposure to heat stress 60°C for 150 s. Cell cultures were previously habituated on lettuce, tomatoes, or in TSB, at 5°C for 24 h and 5 days, and in TSB for 18 h at 30°C (control). Values represent the mean of two independent experiments, with 3 technical replicates each (n = 6).

During heat challenge, cells previously placed on lettuce or in TSB (5°C) for 24 h were more heat-resistant compared to those habituated on tomato (*P* < 0.05), as manifested by *δ* values (Table 6). Both these types of cells were more tolerant than the 5-day habituated cells (*P* < 0.05), also through their higher surviving populations at the end of thermal treatment (N_final_); on lettuce, the survived 24-h stored cells represented 1.80% of the initial population vs. 0.10% for 5-day stored cells. In TSB, the respective N_final_ were 0.28% vs. 0.02% (Fig 4).

**Table 6:** Parameter estimates of Weibull model for inactivation curves during heat stress, carried out in TSB at 60°C, for 150 s.

*Habituation*		Heat challenge at 60°C						
Environment	Conditions	a*	*δ*_*1*_ *(s)*	*p*	*δ*_*2*_ *(s)*	*N*_*res*_ (log CFU/mL)**	*R*^*2*^	*RMSE*
Lettuce	24 h, 5°C	1.6 ± 0.7^A^	43.9 ± 12.2^A^	1.7 ± 0.8^A^	301.0 ± 87.2^A^		0.90 ± 0.14	0.246 ± 0.206
	5 days, 5°C	3.0 ± 1.4^A^	39.4 ± 9.4^AB^	1.1 ± 0.3^AB^	188.5 ± 65.7^A^		0.95 ± 0.04	0.282 ± 0.131
Tomato	24 h, 5°C		22.0 ± 5.1^B^	1.0 ± 0.2^AB^		1.33 ± 0.07	0.92 ± 0.01	0.438 ± 0.032
	5 days, 5°C		20.2 ± 8.5^B^	0.6 ± 0.1^B^			0.90 ± 0.07	0.412 ± 0.151
TSB	24 h, 5°C		43.9 ± 3.8^A^	0.8 ± 0.2^B^			0.88 ± 0.05	0.341 ± 0.070
	5 days, 5°C		41.4 ± 7.1^AB^	0.9 ± 0.2^AB^			0.96 ± 0.01	0.229 ± 0.038
	18 h, 30°C		23.4 ± 3.6^AB^	0.7 ± 0.1^B^			0.96 ± 0.02	0.281 ± 0.068

Values represent mean ± stdev of six replicates. Different letters within the same column indicate significantly different (*P* < 0.05) values of each parameter.

*Modified Weibull model of double sigmoidal inactivation was used for the survival curve of cells habituated on lettuce, for 24 h or 5 days, where *α* = log_10_ (*f* / [1-*f*]), *f* is the fraction of the sensitive subpopulation, and *δ*_*1*_ and *δ*_*2*_ indicate the time needed for the first decimal reduction of sensitive and resistant subpopulation, respectively.

**For the the survival curve of cells habituated on tomatoes for 24 h, Weibull model with curve tailing was used, where *N*_*res*_ represents the residual bacterial population at tail.

*R*^*2*^: regression coefficient

RMSE: Root-mean square error

Since the detection limit during heat challenge was 20 CFU/mL, the *t*_*4D*_ could not be determined.

## Discussion

The results presented here demonstrate that *L*. *monocytogenes* cells previously habituated on fresh lettuce leaves or cherry tomatoes under cold temperatures exhibit a significantly altered response during subsequent exposure to osmotic, acid and heat stresses compared to control cultures grown in synthetic laboratory media. Bacterial cells residing on the external part of produce have to adapt to starvation conditions [18,35], along with the low temperature during storage. *L*. *monocytogenes* cells habituated on tomatoes were the most acid resistant among all treatments tested in the study, and the most sensitized were those habituated in TSB at 5°C. The two mechanisms of acid resistance described by Herbert and Foster [36] included one *sigB*-dependent and one starvation-associated but *sigB*-independent mechanism. Such starvation-induced acid resistance might be the reason of the increased acid tolerance observed in the present study, and the increased acid resistance of the 5-day tomato habituated cells compared to 24-h cells. In addition, the general stress response σ^B^-factor possesses an important role both in maintaining the intracellular pH homeostasis of the pathogen [37] and during its cold acclimation [38,39]. Therefore, investigation of the role of σ^B^-factor in pathogen habituation on fresh produce at cold temperature and in subsequent stress response should be carried out. As opposed to this increased acid resistance, prolonged incubation of *L*. *monocytogenes* EGD-e strain on parsley leaves resulted in increased sensitivity to acetic acid (pH 4.0); nevertheless the incubation on parsley was carried out under 18 and 25°C [14], which is significantly higher compared to the storage temperature used in the current study, and which might have induced the stress response mechanisms. The different acid resistance levels induced on tomato compared to lettuce could be attributed to the different nutrient availability; the thick epicuticular waxy layers on whole tomato surfaces and the tightly packed cellular structure of the epicarp under the skin inhibit mass transfer and water permeability through the tomato skin [40]. On the other hand, cut edges or damaged tissues of lettuce leaves may lead to luck of the protective waxy cuticle, possibly providing epiphytic bacteria with nutrients leaking along the edges [41].

At extracellular pH 3.5, the highest bactericidal activity was exhibited by acetic acid, followed by lactic acid, while HCl had the least effect on cells inactivation. This is most likely attributed to different mode of action of organic and inorganic acids [42], linked to the different pK_a_ of organic acids and their effect on pH homeostasis of the cells [37,43,44]. Indeed, at pH 3.5 the undissociated acid concentration was 0.59 M for acetic acid and 0.08 M for lactic acid. Consequently, at the same external pH value, acetic acid may result in greater lowering of the intracellular pH and hence more severe cellular damage than the lactic acid. Comparing the cells habituated in TSB, the stationary-phase cells (30°C) were more acid-tolerant under all tested conditions than the cells in lag phase (5°C, 24 h) or the early exponential phase cells (5°C, 5 days). Exponential-phase cultures are considered more fragile or require adaptation at a suboptimal pH around 5.0 to 5.5 so as to express acid tolerance, while stationary-phase cultures are naturally acid resistant [44,45].

Tomatoes and lettuce habituation of *L*. *monocytogenes* also resulted in significantly increased tolerance against osmotic stress compared to TSB. In order to withstand low temperature conditions, *L*. *monocytogenes* cells deploy adaptation mechanisms, such as the expression of cold shock proteins (CSPs) and cold acclimation proteins (CAPs) [46], changes in membrane lipid composition, and uptake of osmolytes and oligopeptides [25,47,48]. Of these, uptake of osmolytes is considered the main and universal osmotic stress response mechanism [42,47,49], with carnitine and glycine betaine most commonly used by *L*. *monocytogenes* against harsh cold or osmotic conditions. These low-molecular-weight organic compounds can accumulate to high intracellular concentration without disrupting vital cellular processes [48,50] and occur naturally in foods of animal and plant origin, respectively [51]. The protective effect of glycine betaine on *L*. *monocytogenes* survival on parsley and coleslaw was previously shown [52,53]. Furthermore, the principal role of the alternative σ^B^-factor during growth of cold-stressed *L*. *monocytogenes* was to modulate the accumulation of compatible solutes [38,39], demonstrating the importance of osmolytes in such processes. Therefore, a possible uptake of osmolytes might induce the observed osmotolerance after cold habituation of *L*. *monocytogenes* on fresh produce. Prolonged habituation on tomato resulted in increased osmotolerance, possibly attributed to greater osmolytes accumulation or to the induction of CAPs proteins during the cold storage. The exact reason of higher tolerance to NaCl 20% induced by habituation on tomatoes than on lettuce is not known, but could be associated with other stresses encountered on tomato environment resulting in cross-protection of the cells. In addition, interaction of *L*. *monocytogenes* with the native microbiota, which is specific to each product and could facilitate the uptake of osmolytes or other metabolites by the pathogen [18,19], may also have contributed to the aforementioned phenotype. Research is needed in order to specify the difference in osmolytes uptake from different produce products and the role of incubation time on their accumulation, as higher osmotolerance after prolonged habituation was observed.

In contrast, *L*. *monocytogenes* cells habituated on lettuce or TSB for 24 h were more tolerant during heat challenge at 60°C compared to tomato-habituated cells. Studies have shown that starvation likely results in *L*. *monocytogenes* increased heat resistance [13,36]. On the other hand, growth temperature affected injury and death of *L*. *monocytogenes* exposed to heat [54], and cold shock induced its thermal sensitivity [55]. Our findings showed that habituation in a tomato or lettuce environment was not sufficient to provide the cells with appropriate defenses against heat. This variability in stress response among studies might be also associated with the corresponding strain heterogeneity in these studies [56]. Prolonged acclimation at 5°C (5 days vs. 24 hours) increased thermal sensitivity regardless of the habituation matrices (tomatoes, lettuce or TSB), and these results suggest that cold sensitization might be used to reduce prevalence of *L*. *monocytogenes* in foods subjected to heating.

Overall, it was previously shown that incubation of *L*. *monocytogenes* on parsley leaves enhanced pathogen sensitivity to acetic acid, affected its adherence abilities to abiotic surfaces and reduced its virulence-associated characteristics [14]. Further, investigating here the impact of fresh produce environment on *L*. *monocytogenes*, we demonstrate that habituation on lettuce or tomatoes under cold conditions may provide pathogen cells with increased acid or osmotic stress tolerance. Prolonged habituation on fresh produce along with the cold acclimation (5 days vs. 24 h) may enhance these stress tolerance responses, which is of great importance as habituated cells become more resistant and could be involved in food processing survival and spread of foodborne illness. A diverse growth behavior of *L*. *monocytogenes* on fresh produce leaves is demonstrated, influenced by whether the leaves are in their cut or intact form [41,57–59]. In the present study, we examined the impact of *L*. *monocytogenes* habituation on cut lettuce leaves, based on the hypothetical scenario that cut produce leaves might be involved in cross-contamination during the fresh produce processing and subsequently stored at cold temperatures. Therefore, we refer to a post-harvest contamination event of lettuce or tomato rather than contamination in the field. Pathogens growing in the field and fitting to plant environments, in addition to osmotic, starvation and matric stresses, undergo solar radiation and extreme fluctuations in the physicochemical environment of the phyllosphere over short time scales, as well as plant-microbe interactions and inter-strain interactions [18,19]. In order to survive, adaptive responses are induced, potentially leading to pathogens establishment as a biofilm community or to dominance of persistent strains or subpopulations, further involved in illness transmission. Thus, it is crucial to examine the physiology of pathogenic bacteria on produce and the mechanisms lying behind these phenotypes, in order to develop more efficient strategies to ensure consumers’ safety. Furthermore, the effect of deeper cold acclimation of *L*. *monocytogenes* cells prior to produce inoculation should be evaluated. Finally, the results indicate that as realistic (i.e., closely resembling plant environment) as possible environmental scenarios rather than synthetic laboratory media should be applied in studies assessing the risk associated with behavior of pathogens on fresh produce along the farm-to-fork continuum. This is imperative to avoid underestimation of the pathogen dynamics in fresh produce.
